# Diversity and genomics of giant viruses in the North Pacific Subtropical Gyre

**DOI:** 10.3389/fmicb.2022.1021923

**Published:** 2022-11-25

**Authors:** Roxanna Farzad, Anh D. Ha, Frank O. Aylward

**Affiliations:** ^1^Department of Biological Sciences, Virginia Tech, Blacksburg, VA, United States; ^2^Center for Emerging, Zoonotic, and Arthropod-Borne Infectious Disease, Virginia Tech, Blacksburg, VA, United States

**Keywords:** giant viruses, marine viruses, station ALOHA, *Prasinoviridae*, *Mesomimiviridae*, *Nucleocytoviricota*

## Abstract

Large double-stranded DNA viruses of the phylum *Nucleocytoviricota,* often referred to as “giant viruses,” are ubiquitous members of marine ecosystems that are important agents of mortality for eukaryotic plankton. Although giant viruses are known to be prevalent in marine systems, their activities in oligotrophic ocean waters remain unclear. Oligotrophic gyres constitute the majority of the ocean and assessing viral activities in these regions is therefore critical for understanding overall marine microbial processes. In this study, we generated 11 metagenome-assembled genomes (MAGs) of giant viruses from samples previously collected from Station ALOHA in the North Pacific Subtropical Gyre. Phylogenetic analyses revealed that they belong to the orders *Imitervirales* (*n* = 6), *Algavirales* (*n* = 4), and *Pimascovirales* (*n* = 1). Genome sizes ranged from ~119–574 kbp, and several of the genomes encoded predicted TCA cycle components, cytoskeletal proteins, collagen, rhodopsins, and proteins potentially involved in other cellular processes. Comparison with other marine metagenomes revealed that several have broad distribution across ocean basins and represent abundant viral constituents of pelagic surface waters. Our work sheds light on the diversity of giant viruses present in oligotrophic ocean waters across the globe.

## Introduction

Nucleo-cytoplasmic large DNA viruses (NCLDVs, phylum *Nucleocytoviricota*), also known as “giant viruses,” are a lineage of eukaryotic viruses that include many animal and protist pathogens. In addition to several well-known families that infect vertebrates (e.g., *Poxviridae*, *Asfaviridae*, and *Iridoviridae*), several families in this phylum infect a variety of algae and other protists (e.g., *Phycodnaviridae, Marseilleviridae,* and *Mimiviridae*; Fischer, 2016; Wilhelm et al., 2016; Koonin and Yutin, 2019; Karki et al., 2021; Aylward and Moniruzzaman, 2022). Appreciation of the environmental prevalence of viruses within the *Nucleocytoviricota* somewhat lagged behind other viral groups because the large capsid sizes of many members of this phylum often precluded their recovery in diversity surveys that focused on particles that could pass through a 0.2 μm filter. Nevertheless, pioneering studies focusing on marker genes provided early evidence that these large DNA viruses are widespread in the environment (Chen et al., 1996; Short and Suttle, 2002; Short, 2012), and later metagenomic studies revealed an enormous diversity in this group, particularly in marine environments (Yau et al., 2011; Hingamp et al., 2013; Bäckström et al., 2019; Endo et al., 2020; Moniruzzaman et al., 2020a; Schulz et al., 2020). Recent estimates suggest that there are at least 32 different families of giant viruses that reside in diverse ecosystems across the globe, and more will almost certainly be identified in the future (Aylward et al., 2021).

Giant viruses have complex and chimeric genomes that are the product of widespread gene exchange with various cellular lineages (Boyer et al., 2009; Yoshikawa et al., 2019; Moniruzzaman et al., 2020a,b). Besides the core machinery involved in virion structure and DNA replication, giant viruses also commonly encode genes involved in translation, glycolysis, TCA cycle, cytoskeletal dynamics, light-harvesting, nutrient transport, and other pathways involved in nutrient homeostasis (Schulz et al., 2017; Moniruzzaman et al., 2020a). Rhodopsins are also common in a wide range of marine giant viruses (Yutin and Koonin, 2012; Needham et al., 2019; Rozenberg et al., 2020). Rhodopsins are light-driven ion pumps that can be involved in energy production and signal transduction (Ernst et al., 2014; Govorunova et al., 2017). Viral rhodopsins may permit viruses to modify host phototaxis during infection, which may promote their proliferation (Gallot-Lavallée and Archibald, 2020). Proteins involved in cytoskeletal dynamics have also been found to be quite common in a variety of marine giant viruses; viral homologs to actin, myosin, and kinesin genes could potentially benefit viruses by manipulating the host’s cytoskeleton by using host motor proteins to traffic virions or maintain the localization of viral machinery during infection (Ha et al., 2021; Kijima et al., 2021; Da Cunha et al., 2022). These recent findings collectively suggest that giant viruses use a broad assortment of functional genes to manipulate host physiology and alter the intracellular environment to promote virion propagation.

Although giant viruses are globally distributed in a variety of habitats, they appear to be particularly diverse and abundant in the ocean (Ghedin and Claverie, 2005; Monier et al., 2008; Endo et al., 2020; Moniruzzaman et al., 2020a). The majority of the ocean is made up of oligotrophic oceanic gyres, and it is therefore of particular interest to examine viral dynamics in these systems. One field site that has been particularly useful for examining microbial diversity in oceanic gyres is Station ALOHA (A Long-term Oligotrophic Habitat Assessment), located at 22°45′N, 158°W, nearly 100 km north of the Hawaiian island of Oahu (Karl and Church, 2014). Several recent studies have recently elucidated a rich diversity of viruses that are present at or near Station ALOHA (Aylward et al., 2017; Luo et al., 2017, 2020, 2022). In this study, we surveyed previously-sequenced metagenomes generated from Station ALOHA to characterize the diversity of giant viruses in this habitat (Mende et al., 2017). Although metagenomes derived from <0.2 μm size fractions are typically used to evaluate viral diversity in marine systems, recent studies have found that many giant viruses are often found in larger size fractions along with bacteria and archaea (Endo et al., 2020; Moniruzzaman et al., 2020a). We also analyzed the encoded functions in the draft giant virus genomes that we recovered to gain insight into possible mechanisms they employ to manipulate their hosts during infection. Lastly, by examining publicly-available metagenomes from other marine environments we examined the distribution and biogeography of these viruses on a global scale. Because of Station ALOHA’s location in the North Pacific Subtropical Gyre, examination of giant viruses found here provides a window into those lineages that are likely broadly distributed in oligotrophic ocean waters and may play important roles in marine ecological dynamics.

## Materials and methods

### Metagenomes used

We analyzed metagenomes that were generated in a previous study (Mende et al., 2017). This dataset consists of 107 samples from depths ranging from 25 m to 1,000 m that were collected over a 1.5 year sampling period at Station ALOHA on 11 cruises of the Hawaii Ocean Time-series (HOT). The methods used for sample collection and processing have been previously described (Mende et al., 2017). Briefly, water was filtered onto 0.2 μm filters, and after DNA extraction, libraries were created with Illumina TruSeq LT Nano kit, and metagenomes were sequenced using Illumina MiSeq and NextSeq 500 systems.

### Generation of metagenome-assembled genomes

MAGs were generated using a workflow developed previously (Aylward and Moniruzzaman, 2021). Briefly, metagenomes were assembled with MegaHit v. 1.2.9 (with parameters –min-contig-len 5000), and contigs were subsequently binned using MetaBat2 v. 2.12.1 (with parameters-s 100000, -m 10000, -minS 75, -maxEdges 75; Kang et al., 2019). All bins were analyzed with ViralRecall v. 2.0 to identify those that corresponded to giant viruses, and only those that contained 4 of 5 NCLDV marker genes were retained. The marker genes used for this were the A32-like ATPase (A32), B-family DNA polymerase (PolB), virus late transcription factor 3 (VLTF3), major capsid protein (MCP), and superfamily II helicase (SFII). This resulted in 11 NCLDV bins ranging in size from 119,690 to 574,081 bp (Table 1). Genome statistics were compiled with SeqKit v. 2.2.0 (Shen et al., 2016), and we predicted proteins using Prodigal v. 2.6.3 (Hyatt et al., 2010) with default parameters (Table 1).

**Table 1:** General characteristics of 11 metagenome-assembeled genomes (MAGs) of giant viruses generated from St. ALOHA.

Genome	Genome length	GC content %	Num of protein coding genes	Order	Family	Genus
HOT_MAG4	122,808	38.47	164	*Algavirales*	AG_04	g175
HOT_MAG14	147,238	37.49	200	*Algavirales*	AG_01	g177
HOT_MAG30	171,883	34.21	260	*Algavirales*	AG_01	g177
HOT_MAG20	119,690	33.82	174	*Algavirales*	AG_01	g177
HOT_MAG12	386,441	33.29	412	*Imitervirales*	IM_01	g336
HOT_MAG13	433,885	32.35	475	*Imitervirales*	IM_01	g342
HOT_MAG10	426,436	29.37	491	*Imitervirales*	IM_01	g342
HOT_MAG3	574,081	31.33	559	*Imitervirales*	IM_09	g279
HOT_MAG5	477,804	30.13	484	*Imitervirales*	IM_09	g279
HOT_MAG60	489,708	24.69	541	*Imitervirales*	IM_09	g274
HOT_MAG22	471,006	34.86	484	*Pimascovirales*	PM_01	NA

### Phylogenetic construction

**Figure 1:**
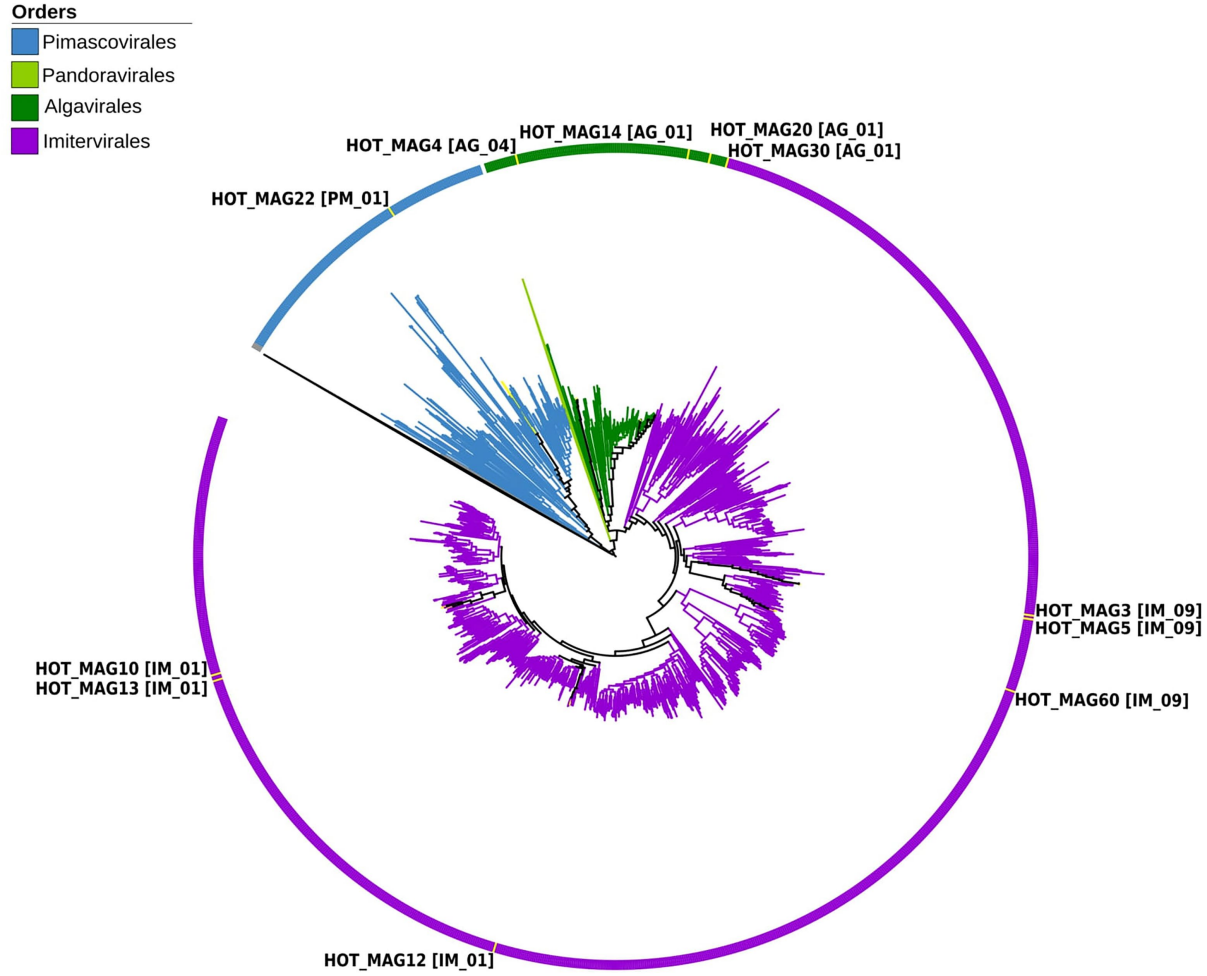
Multi-locus phylogenetic tree of the 11 MAGs together with 1,381 reference genomes from the Giant Virus Database. The phylogenetic tree was constructed using 7 maker genes that are highly conserved in giant viruses (see Materials and methods for details). According to the constructed phylogenetic tree, HOT_MAG22 belongs to the order *Pimascovirales* [PM_01]. HOT_MAG4 [AG_04], HOT_MAG14 [AG_01], HOT_MAG20 [AG_01] and HOT_MAG30 [AG_01] clustered within the order *Algavirales*. The rest of the MAGs, HOT_MAG3 [IM_09], HOT_MAG5 [IM_09], HOT_MAG60 [IM_09], HOT_MAG12 [IM_01], HOT_MAG13 [IM_01] and HOT_MAG10 [IM_01] belong to the order *Imitervirales*.

In order to explore the phylogenetic placement of the reconstructed MAGs, we used the protein predictions of the 11 MAGs generated in this study together with proteins from all reference giant viruses that have previously been compiled in the Giant Virus Database (Aylward et al., 2021). Subsequently, we used the program ncldv_markersearch to generate a concatenated alignment of 7 marker genes, as previously described^1^ (Aylward et al., 2021). Briefly, this tool use HMMER3 to identify 7 conserved marker genes (superfamily II helicase (SFII), virus-like transcription factor (VLTF3), B-family DNA polymerase (PolB), and A32-like ATPase (A32), a DNA-dependant RNA polymerase (RNAP) subunit, transcription elongation factor II-S (TFIIS), and a family II topoisomerase), and then uses Clustal Omega v1.2.4 to produce multi-sequence alignments, which are then concatenated. Proteins that are absent in a MAG are replaced with a series of X characters in the concatenated alignment. We then generated a maximum-likelihood phylogenetic tree using IQ-TREE v. 1.6.12 with 1,000 ultrafast bootstraps (parameters-m LG + F + I + G4-bb 1,000-wbt -nt AUTO -runs 3; Hoang et al., 2018; Minh et al., 2020). The tree was visualized in the interactive Tree of Life (iTOL; Letunic and Bork, 2019; Figure 1).

### Average nucleotide and amino acid identity

In order to assess divergence from reference genomes, we calculated one-way average amino acid identity (AAI) and average nucleotide identity (ANI) of the 11 MAGs against all genomes in the Giant Virus Database. Both comparisons were done with LAST v. 959, and results were parsed with a custom Python script. Results are shown in Supplementary Table S1 (Kiełbasa et al., 2011).

### Sequence similarity search and protein annotation

**Figure 2:**
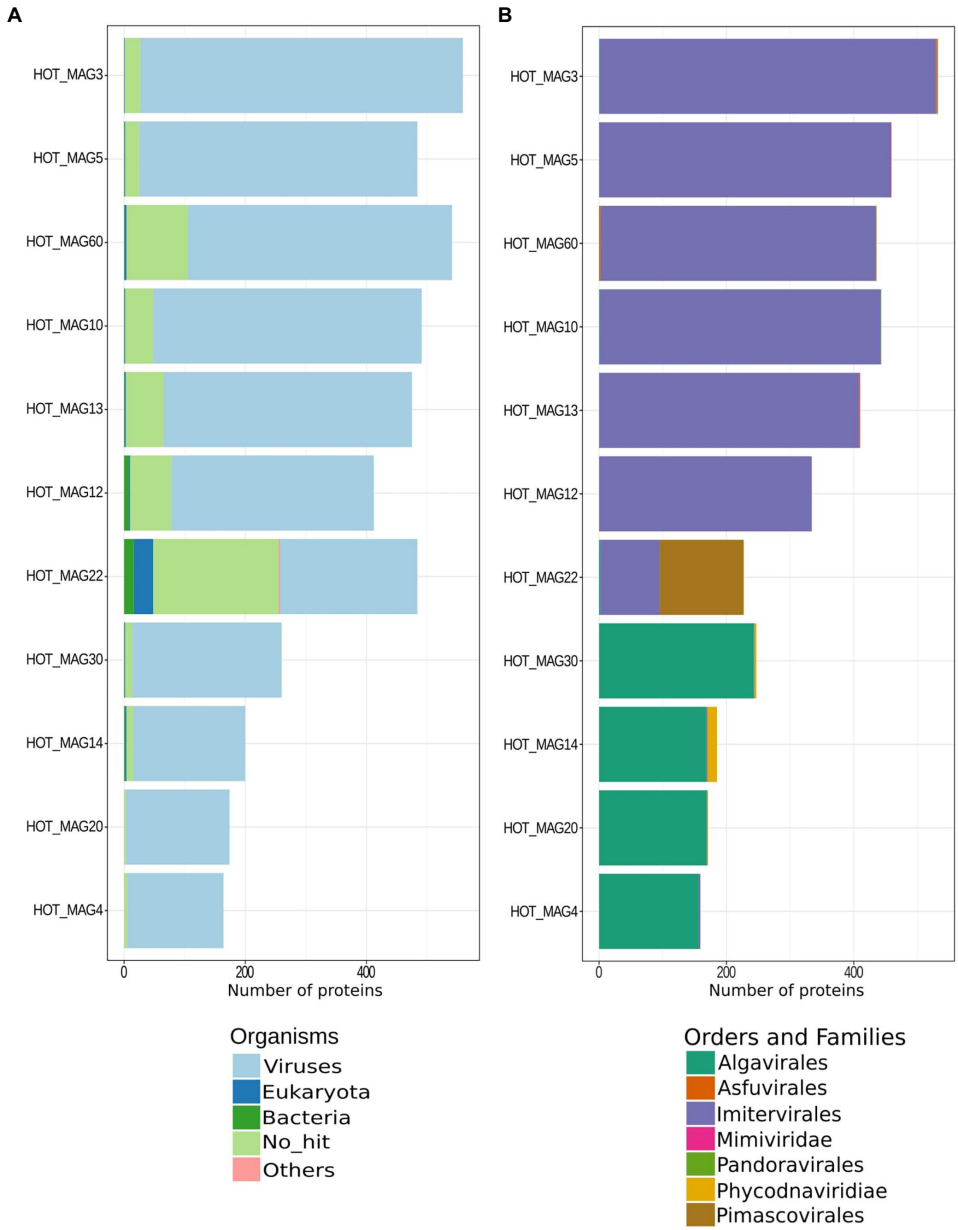
Best matches of predicted proteins identified in each HOT MAG. The results are based on a LAST-based homology search, with best hits retained (see Materials and methods for details).

To perform sequence homology searches, we used LAST v. 959 (parameters-m 5000, -f BlastTab, -P 32, -u 2, -Q 0) to compare all protein predictions against a protein database that included RefSeq 207 as well as all protein predictions in the Giant Virus Database (O’Leary et al., 2016; Aylward et al., 2021). We indicated the sequence similarities between 11 MAGs and multiple organisms (bacteria, eukaryotes, viruses, and others) together with those which have no hits (Figure 2A). Moreover, we retained all the best hits to viruses and searched for homology between MAGs and different viral orders and families (Figure 2B). Subsequently, plots to visualize the results were made with ggplot2 v. 3.3.6 in R software (Wickham, 2011; Figure 2) and the final results of the sequence similarity search are accessible in Supplementary Table S2. For protein functional prediction, we annotated all predicted proteins in each genome by searching them against the Pfam database v. 34 (Nguyen et al., 2011) using HMMER3 v. 3.3 (parameter “–cut_nc”) with all hits retained. These annotations are available in Supplementary Table S3. Protein annotations were manually inspected to detect the presence of genes involved in central carbon metabolism, DNA processing, light harvesting, amino acid metabolism, cytoskeleton dynamics, and other functions of interest.

### Read-mapping analysis

We examined the distribution of our 11 MAGs by mapping the reads from other marine metagenomes onto them using coverM 0.6.1 (parameters –min-read-percent-identity 0.95, minimum 20% covered fraction; available from https://github.com/wwood/CoverM). We used a breadth cutoff of 20% (i.e., 20% covered fraction), consistent with a recent study that used read-mapping to determine the distribution of large bacteriophages (Weinheimer and Aylward, 2022). We used the metagenomic datasets corresponding to the GA02, GA03, GP13, and GA10 cruises from the bioGEOTRACES dataset (Biller et al., 2018). These metagenomes were sequenced from 480 samples collected during 2010–2011 from 91 stations. The location and the sampling date of each of the transects are as follows: GA02 (from North to South Atlantic, May 2010–March 2011), GA03 (North Atlantic, October 2010–December 2011), GA10 (South Atlantic, October 2010–November 2010), GP13 (South Pacific Ocean, May 2011–June 2011). In addition, we evaluated the distribution of 11 MAGs in St. ALOHA across different depths (0–1,000 m) and across a 1.5-year time series at that location to evaluate their depth distribution and seasonal abundance in this oligotrophic ecosystem. The Hawaii Ocean Time-series (HOT) metagenomes were previously generated (Mende et al., 2017) during approximate monthly sampling periods from August 2010 to December 2011 at St. ALOHA. Reads from these metagenomes were mapped onto the 11 MAGs using the same coverM parameters as described above. For visualization, we used R and R-based tools (v. 3.3.2)^2^ to draw world map plots and Ocean Data View software (v. 4.7.10)^3^ for contour and Hawaii Ocean Time-series (HOT) plots. Depth distribution was interpolated in Ocean Data View in the DIVA gridding mode. The raw read mapping statistics of 11 MAGs on a global scale and in Station ALOHA are available in Supplementary Tables S4 and S5, respectively.

## Results and discussion

### Phylogenetic analysis of the giant virus metagenome-assembled genomes

Based on our multi-locus phylogenetic analysis, four of the MAGs can be placed within the order *Algavirales* (HOT_MAGs 4, 14, 20, and 30), six could be assigned to the *Imitervirales* (HOT_MAGs 3, 5, 60, 12, 13, and 10), and one could be placed in the *Pimascovirales* (HOT_MAG 22; Figure 1). Among the MAGs that fall within the *Algavirales*, MAGs 14, 30, and 20 belong to the family *Prasinoviridae* [AG_01] and the same genus-level group (g177) (Table 1). Viruses in this family are known to infect prasinophytes of the genera *Bathycoccus*, *Micromonas*, and *Ostreococcus* (Weynberg et al., 2017). Prasinophytes are picoeukaryotic algae that are broadly distributed in the ocean (Moreau et al., 2010; Lopes dos Santos et al., 2016). Prasinoviruses are correspondingly abundant in marine waters and play a crucial role in regulating the populations of their plankton hosts (Bellec et al., 2010). Cultivated representatives of these viruses have genome sizes ranging from 184 to 198 kbp and %GC contents from 37% to 45%. This is generally consistent with the prasinovirus MAGs that we recovered here, which range in size from ~120–172 kbp and in %GC content from 33.8%–37.5% (Table 1). The somewhat smaller size of HOT_MAGs 14 and 20 is potentially an indication that these genomes are not complete, though it may also be a sign of genome reduction compared to their relatives. The last MAG that could be placed within the *Algavirales*, HOT_MAG 4, clustered within the family-level lineage AG_04 and the genus g175, both of which were recently demarcated (Aylward et al., 2021; Figure 1; Table 1). The sole cultivated representative of this lineage is *Heterosigma akashiwo virus* (HaV), which infects the eponymous raphidophyte that is responsible for causing harmful algal blooms (Nagasaki et al., 2005). Although raphidophytes are commonly associated with algal blooms in coastal waters, they have also been reported in oligotrophic gyres (Leles et al., 2019), and it is, therefore, possible that the host of this MAG lies within this group.

Of the six *Imitervirales* MAGs, three fall within the proposed *Mesomimiviridae* family (IM_01: HOT_MAGs 10, 12, 13) (Figure 1; Table 1). HOT_MAGs 10 and 13 are clustered within the same genus g342 while HOT_MAG12 falls within the genus g336 (Table 1). Members of the *Mesomimiviridae* are particularly widespread in global marine and freshwater environments, and phylogenomic analysis suggests that roughly half of all currently available MAGs can be placed in this group (Aylward et al., 2021). The size of these MAGs ranges from 380 to 430 kbp (Table 1), which is consistent with those of cultivated viruses in this family that infect haptophyte hosts of the genera *Chrysochromulina* and *Phaeocystis* (Gallot-Lavallée et al., 2017). *Phaeocystis globosa* virus 16T (PgV-16T) is one of the most well-studied members of this family; it has a 150 nm diameter icosahedral virion with a 470 kbp genome size, which is comparable to the related viruses that infect *Chrysochromulina ericina* virus *C. parva* (Gallot-Lavallée et al., 2017; Stough et al., 2019).

The last three *Imitervirales* MAGs clustered within the recently demarcated family IM_09 (Figure 1; Table 1), which contains *Aureococcus anophagefferens* virus (AaV) and *Prymnesium kappa* virus (PkV). AaV is the smallest member of the *Imitervirales* isolated to date, with a genome size of 371 kbp and a virion diameter of 140 nm (Moniruzzaman et al., 2014; Gann et al., 2020). In contrast to this, PkV is rather large, with a 1.4 Mbp genome and a ~310 nm diameter virion (Johannessen et al., 2015; Blanc-Mathieu et al., 2021), underscoring the large range of genome and virion sizes within this group. HOT_MAGs 3 and 5 fall within the same genus g279 while HOT_MAG 60 clustered within genus g274. The three MAGs that fall within this group have genome sizes ranging from 470 to 575 kbp (Table 1), which is larger than AaV but quite a bit smaller than PkV, suggesting that they represent intermediate-sized members of this lineage.

The only MAG that clustered within the *Pimascovirales* is HOT_MAG 22, which falls within the recently demarcated family-level lineage PM_01 (Figure 1; Table 1). Interestingly, HOT_MAG 22 falls outside of previously demarcated genus-level groups and had low AAI to any references (highest match of 40% AAI to GVMAG-M-3300023184-117, Supplementary Table S1), suggesting that this MAG represents a new genus-level group. The order *Pimascovirales* includes several lineages that infect amoeba, such as Pithoviruses and Marseilleviruses, as well as other viruses that infect metazoan hosts ranging from insects to fish and frogs, such as the *Iridoviridae/Ascoviridae*. Pimascoviruses are not commonly viewed as widespread marine viruses, although, in the previous studies, other MAGs that fall within the PM_01 group have also been found in marine environments (Aylward et al., 2021), and in some cases have been found to be transcriptionally active (Ha et al., 2021). One study also reported several Pimascovirus MAGs from deep sea sediments, including one with phylogenetic placement near the Marseilleviruses and a notably large genome (>700 kbp; Bäckström et al., 2019). The prevalence of these viruses in marine metagenomes suggests that they infect as-yet unknown protist hosts, and further research identifying their host range will be a necessary step toward clarifying the ecological impacts of marine pimascoviruses.

The phylogenetic placements of the 11 reconstructed MAGs are in agreement with the result from the homology search results of all proteins encoded by each MAG. Proteins in each MAG had best matches to viruses in the same order in which they were classified in the phylogeny (Figure 2). The only possible exception is the *Pimascovirales* MAG (HOT_MAG 22) which had a larger proportion of proteins with no known homologs or hits to the *Imitervirales*. This may be due to the relatively poor representation of marine *Pimascovirales,* together with the large number of *Imitervirales* in our reference genome databases.

### Environmental distribution of the giant virus metagenome-assembled genomes

**Figure 3:**
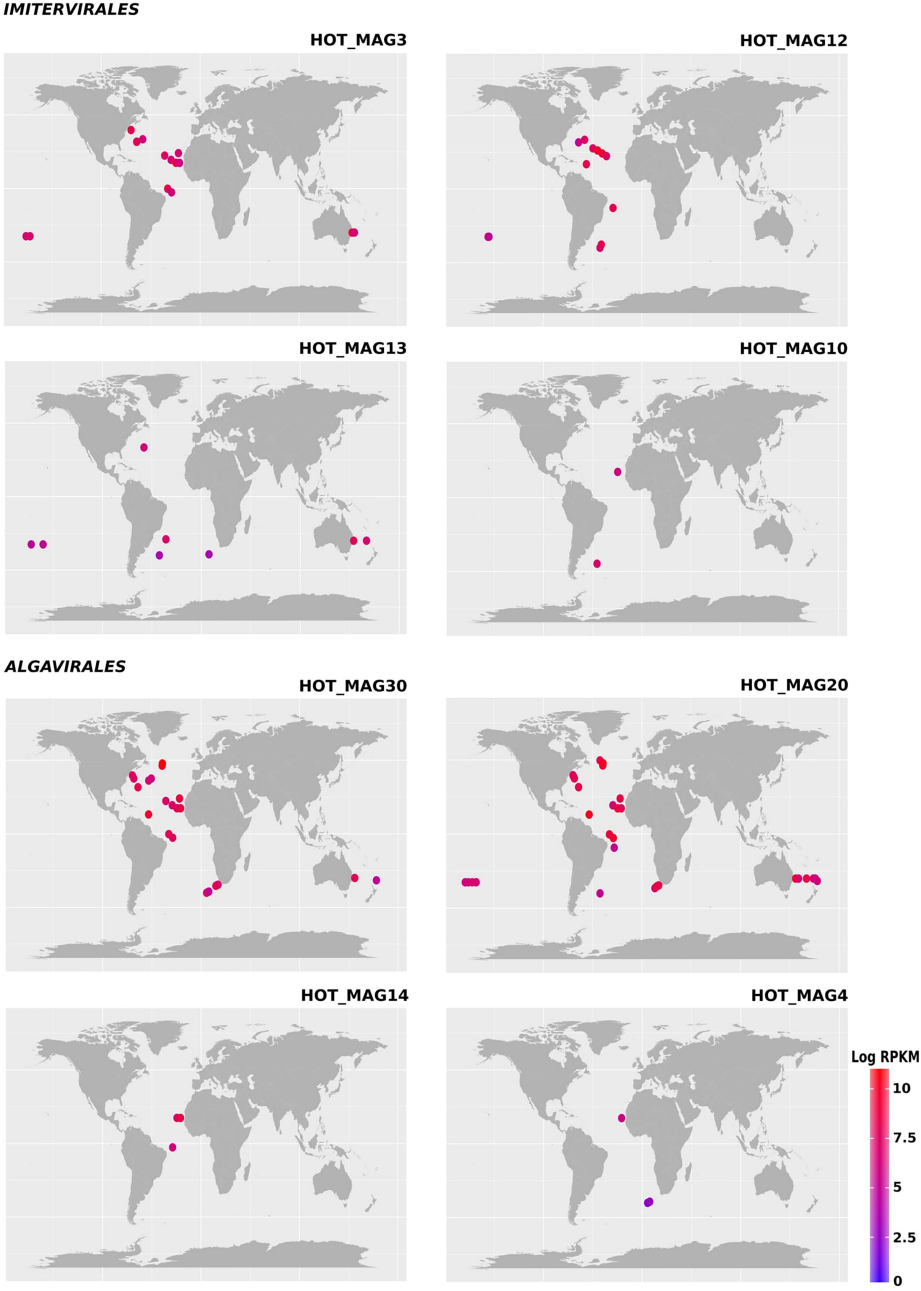
Environmental distribution of the HOT MAGs based on read-mapping of bioGEOTRACES metagenomes. HOT_MAGs (22,60) are not shown as they were not identified in bioGEOTRACES metagenomic dataset. HOT_MAG5 is not presented as it is closely related to HOT_MAG3 and its abundance is nearly the same as HOT_MAG3. Bubbles indicate the global distribution of the MAGs which was calculated based on RPKM. Moreover, the abundance of the MAGs in distinct sites with the same latitude and longitude are reported in parenthesis; HOT_MAG3 (15), HOT_MAG12 (12), HOT_MAG13 (8), HOT_MAG10 (2), HOT_MAG30 (23), HOT_MAG20 (30), HOT_MAG14 (3), and HOT_MAG4 (3).

Previous studies have shown that the proposed *Mesomimiviridae* family is particularly prevalent in aquatic systems (Santini et al., 2013; Stough et al., 2019; Aylward et al., 2021). Consistent with this, we found that the three MAGs that fall within this clade were particularly widespread in the marine metagenomes that we surveyed here (HOT_MAGs 12, 13, and 10; Figure 3). HOT_MAGs 12, 13, and 10 were found in 12, 8, and 2 bioGEOTRACES sample locations, respectively. HOT_MAG 3, which can be classified into the family-level group IM_09, was detected in 15 distinct locations and is the most broadly-distributed of the MAGs that fall within the *Imitervirales* MAGs. HOT_MAGs 20 and 30, which fall within the *Prasinoviridae* family, were the most abundant MAGs in the bioGEOTRACES datasets (Figure 3). HOT_MAGs 20 and 30 were also quite widespread, occurring in 23 and 30 distinct locations, respectively. The third prasinovirus (HOT_MAG 14), and the other MAG that places within the *Algavirales* (HOT_MAG4, family AG_04) were each found in only 3 sample sites within the Atlantic Ocean (Figure 3). It is worth noting that HOT_MAGs 60 and 22 were not found in any reference bioGEOTRACES metagenomic dataset. HOT_MAG60 can be placed within the order *Imitervirales* (family IM_09) while HOT_MAG22 is the only member of the *Pimascovirales* we identified. This suggests that these viruses are either quite rare or typically found in low relative abundances and cannot be readily detected with metagenomic methods. Although these HOT_MAGs could only be detected at Station ALOHA, it seems unlikely that they are endemic to this region given that the prevailing conditions at this station are representative of widespread oligotrophic waters.

**Figure 4:**
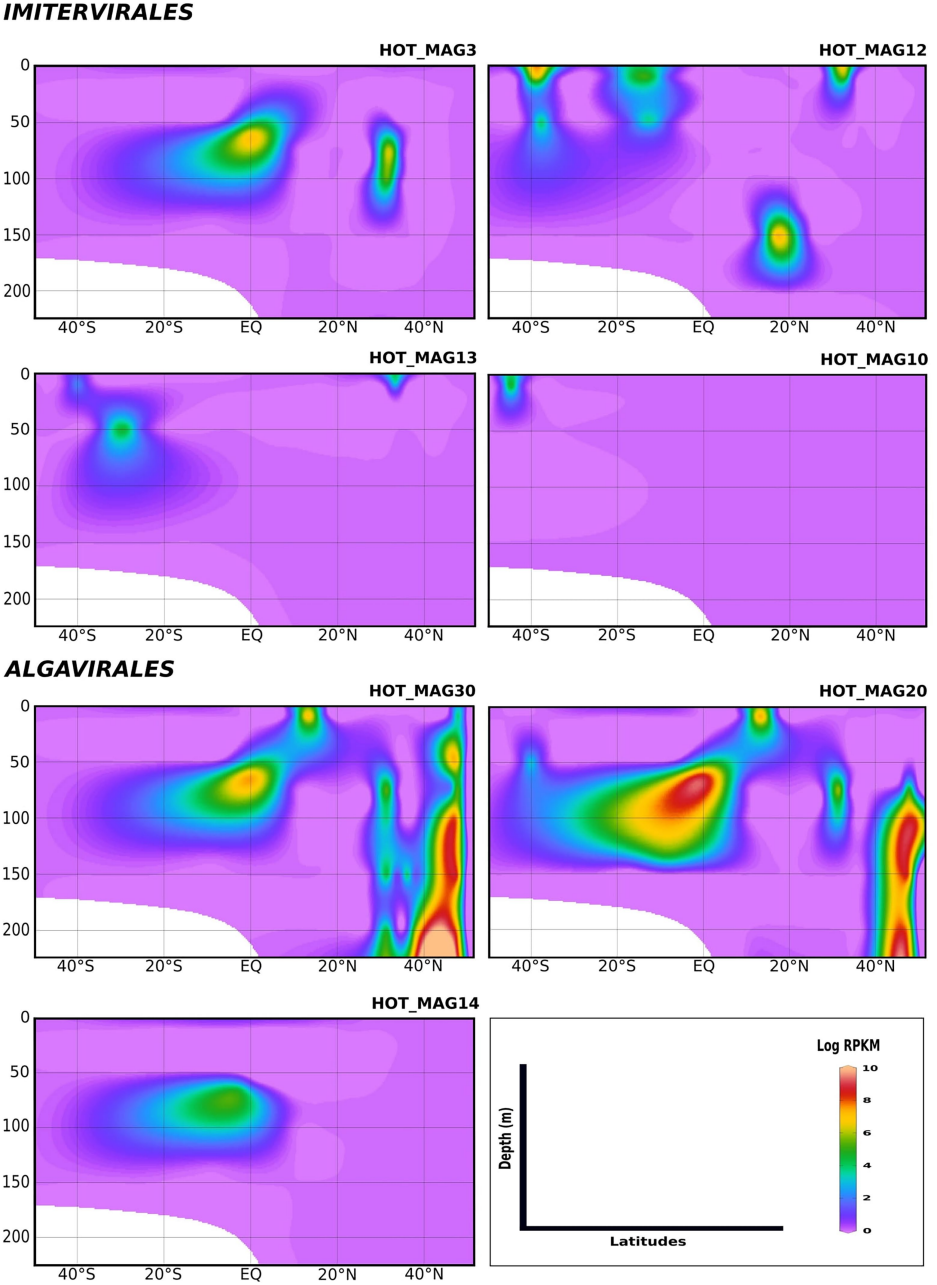
Environmental distribution of the HOT MAGs across a depth profile of the GA02 transect (North Atlantic to South Atlantic). The contour plots were drawn based on the results from mapping GA02 metagenome dataset onto the 11 MAGs (see Materials and methods for details). The total abundance of the MAGs was calculated based on RPKM. Y-axis and x-axis represent depths(m) and latitudes, respectively and the colorful bar refers to the log of total abundance (RPKM) for each of the MAGs (high abundance = red and low abundance = purple). HOT_MAGs (22, 4, 60) are not shown as they were not found in the GA02 metagenomic dataset. HOT_MAG5 is also not presented as it is closely related to HOT_MAG3 and its abundance is almost the same as HOT_MAG3.

We examined the bioGEOTRACES GA02 transect in more detail because it traverses the north and south Atlantic and therefore allows for examination of trends in viral abundance across both latitude and depth (Figure 4). Many of the MAGs that fall within the *Imitervirales* were mostly concentrated in oligotrophic surface waters (above 100 m), consistent with their initial identification at Station ALOHA. These MAGs were identified across a wide range of latitudes; while HOT_MAG3 showed the highest prevalence near the equator, HOT_MAGs 10, 12, and 13 could be detected at latitudes near 40 degrees. Among the MAGs within the *Algavirales*, we detected only the three *Prasinoviridae* MAGs in the GA02 transect metagenomes. Two of these HOT_MAGs (20 and 30) showed high abundance both in equatorial waters as well as high northern latitudes (Figure 4). In equatorial waters all prasinovirus MAGs were found in waters above 100 m, but in the north Atlantic HOT_MAGs 20 and 30 could be found in waters near 200 m, possibly due to sinking water masses in this area (i.e., overturning circulation).

**Figure 5:**
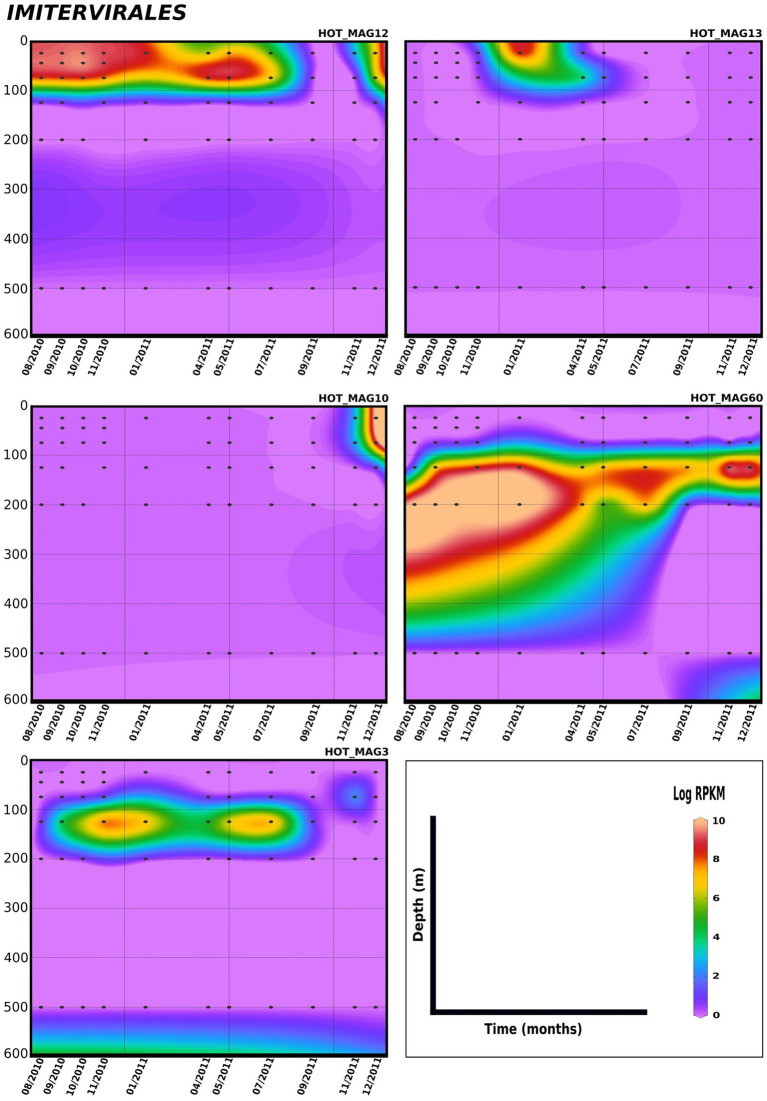
Distribution of 11 MAGs in the order *Imitervirales* in a 1.5-year sampling from St. ALOHA. Time series plots were drawn based on the results from mapping Hawaiian Ocean Time series metagenome dataset onto the 11 MAGs (see Materials and methods for details). The total abundance of the MAGs was calculated based on RPKM. Y-axis and x-axis represent depths(m) and latitudes, respectively and the colorful bar refers to the log of total abundance (RPKM) for each of the MAGs (high abundance = red and low abundance = purple). Only the distribution of the MAGs between 0 and 600 m were shown in the plots.

**Figure 6:**
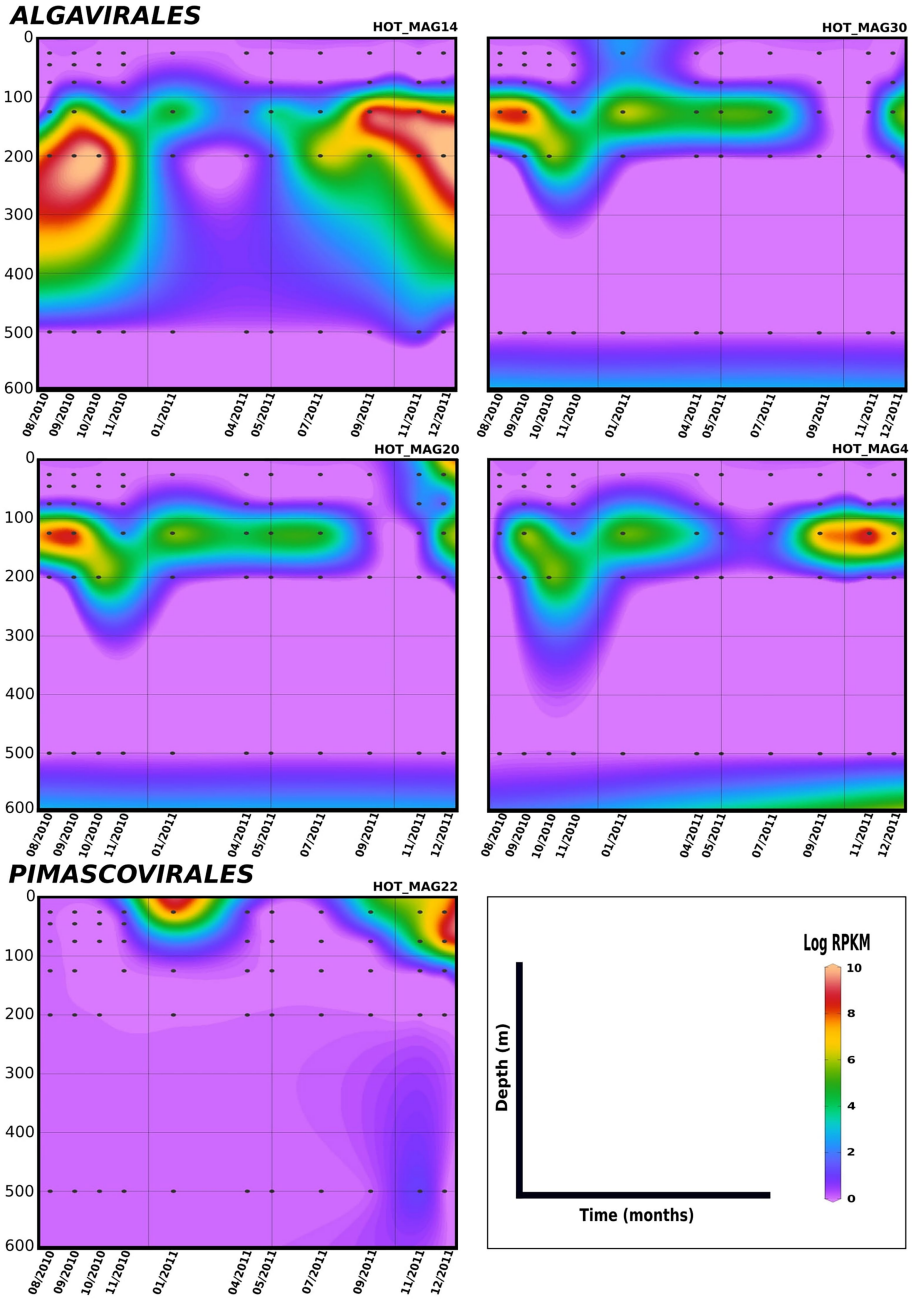
Distribution of 11 MAGs in the orders *Algavirales* and *Pimascovirales* in a 1.5-year sampling from St. ALOHA. Time series plots were drawn based on the results from mapping Hawaiian Ocean Time series metagenome dataset onto the 11 MAGs (see Materials and methods for details). The total abundance of the MAGs was calculated based on RPKM. Y-axis and x-axis represent depths(m) and latitudes, respectively and the colorful bar refers to the log of total abundance (RPKM) for each of the MAGs (high abundance = red and low abundance = purple). Only the distribution of the MAGs between 0–600 m were shown in the plots.

Lastly, in order to evaluate temporal trends in viral abundance, we also examined the presence of the MAGs across a 1.5 year time-series at Station ALOHA from August 2010 to December 2011, which encompasses the same metagenomes used to generate these MAGs (Figures 5, 6). Here the *Algavirales* MAGs seem to be more prevalent than the *Imitervirales* MAGs during the sampling period from St. ALOHA. Among those MAGs that fall within the family *Mesomimiviridae*, all are present in surface waters (above 100 m), and HOT_MAG12 was prevalent throughout almost the entire 1.5 year sampling period (Figure 5). Within *Agavirales* MAGs, two members of the family *Prasinoviridae* (HOT_MAGs 20, 30) have almost the same pattern of their distribution and more likely to be present during August and September, while HOT_MAG14 is abundant in greater depths (100–500 m) and prevalent in almost all seasons except for spring. HOT_MAG4, another *Agavirales* MAG, also has the same distribution trend as HOT_MAGs (20, 30), however, this MAG is highly abundant during November and December (Figure 6). Overall, all of the MAGs within the *Algavirales* were prevalent in the 125 m samples, which was just below the DCM for the majority of cases. HOT_MAG30, HOT_MAG20 and HOT_MAG4 were found most frequently at 125 m, while HOT_MAG14 was also prevalent in several 200 m samples (Figure 6). This transition below the DCM is consistent with the large-scale microbial community turnover that occurs in this region at Station ALOHA (Mende et al., 2017). The only *Pimascovirales* MAG (HOT_MAG22), is distributed in shallow depths (0–100 m) and is highly concentrated between December and January (Figure 6).

### The genomic repertoire encoded in the 11 metagenome-assembled genomes

**Figure 7:**
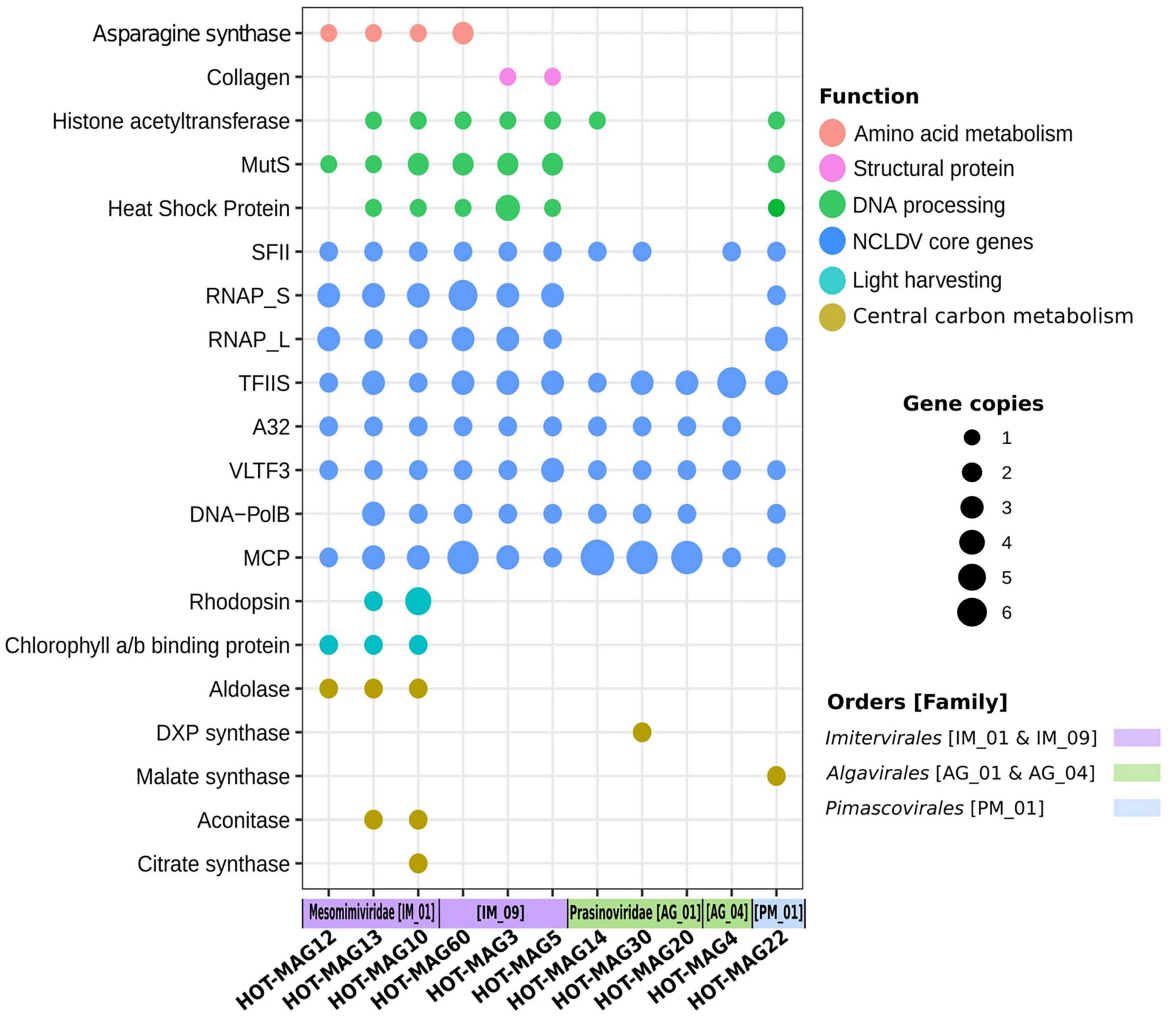
Distribution of selected genes within 11 MAGs of giant viruses. The x-axis indicates the 11 MAGs of giant viruses together with the viral families and orders that they belong to. The size of the bubbles indicate the total abundance of the genes within the genomes of 11 MAGs. the abbreviations of the encoded genes are as follows: MutS: mismatch DNA repair, RNAP: DNA-dependent RNA polymerase Large and Small subunits, SFII: superfamily II helicase, TFIIB: transcription factor IIB, A32: A32 ATPase, VLTF: viral late transcription factor 3, MCP: major capsid protein, DXP synthase: 1-Deoxy-D-xylulose 5-phosphate synthase.

Giant viruses have complex genomes that encode various genes that are not commonly found in viral lineages, including components of central carbon metabolism and translation-associated proteins. As expected, we found giant viruses core genes involved in DNA replication, transcription, and virion structure (Figure 7). We did not find RNAP subunits in the prasinovirus MAGs, consistent with previous studies showing that this lineage lacks this enzyme (Moreau et al., 2010; Figure 7). The absence of RNAP subunits suggests that these viruses have a nuclear stage to their infection in which host RNAP is used for viral gene expression.

Asparagine synthase genes were identified among the *Imitervirales* MAGs, in particular among those members of the *Mesomimiviridae* (Figure 7). This enzyme was previously identified in Phaeocystis globosa virus, though it remains unclear what role it may play during infection (Santini et al., 2013). Collagen encoded genes were only found in *Imitervirales* MAGs belonging to the family IM_09, suggesting that these proteins may be a part of the structure of the virions of these viruses. Among the genes involved in DNA processing, chaperones from the heat shock protein families (HSP70, HSP90) which are presumably used for capsid protein folding (Legendre et al., 2010), DNA mismatch repair (MutS), and histone acetyltransferase known to be functional for packaging DNA within the capsid (Koonin and Yutin, 2010; Legendre et al., 2010), were mostly frequent among *Imitervilares* and *Pimascovirales* MAGs but were mostly absent from the *Algavirales* MAGs (Figure 7).

Many of the MAGs encode multiple genes involved in central carbon metabolism, such as aldolase, 1-Deoxy-D-xylulose 5-phosphate synthase (DXP synthase), malate synthase, aconitase, and citrate synthase. Our findings indicate genes that belong to central carbon metabolism are mostly detected in *Mesomimiviridae* MAGs, notably those that are predicted to be involved in predicted to be involved in the TCA cycle (Figure 7). Interestingly, malate synthase and DXP synthase were only found in the *Pimascovirales* MAG and *Algavirales* MAG, respectively. Previous work has shown that enzymes involved in central carbon metabolism are quite common in many giant viruses (Moniruzzaman et al., 2020a), and our results here are consistent with those findings.

Previous studies have shown that rhodopsins and chlorophyll-binding proteins are quite common in a wide range of marine giant viruses (Yutin and Koonin, 2012; Needham et al., 2019; Moniruzzaman et al., 2020a; Rozenberg et al., 2020). Consistent with this, we found chlorophyll binding proteins in all three mesomimiviruses, and rhodopsin homologs in two (HOT_MAGs 10 and 13) (Figure 7). This suggests that these viruses likely infect phototrophic or mixotrophic hosts and manipulate light harvesting machinery during infection. All mesomimivirus MAGs were detected in shallow waters at Station ALOHA, consistent with the prevalence of their hosts in well-lit surface waters. Interestingly, we detected three rhodopsin homologs in HOT_MAG10; it is unclear what role these three enzymes would play during infection, but the presence of three distinct homologs suggests that they are an important component of the infection strategy of many marine giant viruses.

## Conclusion

Our study sheds light on the phylogenetic diversity, genomics, and distribution of giant viruses in oligotrophic marine waters. We present 11 MAGs of giant viruses that we reconstructed from metagenomes generated from Station ALOHA in the North Pacific Subtropical Gyre. These MAGs fall within five families in the orders *Imitervirales*, *Algavirales*, and *Pimascovirales*. Those MAGs that fall within the *Prasinoviridae* and *Mesomimiviridae* families are the most widespread and abundant, and several of these MAGs were detected in diverse bioGEOTRACES metagenomes that were collected in different ocean basins. Several of the MAGs were found consistently at Station ALOHA over a 1.5 year period, suggesting they are persistent community members in oligotrophic waters. The MAGs encoded a diverse range of functions, including genes involved in central carbon metabolism and light harvesting, suggesting that they use a variety of strategies to manipulate the physiology of their hosts during infection. Our work contributes to a growing body of research that suggests that large DNA viruses are abundant and widespread components of marine systems that play key roles in ecological dynamics and biogeochemical cycling.

## Data availability statement

The datasets presented in this study can be found in online repositories. The names of the repository/repositories and accession number(s) can be found in the article/Supplementary material.

## Author contributions

FA and RF contributed in the conceptualization of the research and writing the manuscript. RF and AH performed bioinformatic analysis of the research. All authors contributed to the article and approved the submitted version.

## Funding

This research was funded by a Simons Foundation Early Career Award in Marine Microbial Ecology and Evolution, NSF CAREER award 2141862, and NIGMS R35 award 1R35GM147290-01 to FA.

## Conflict of interest

The authors declare that the research was conducted in the absence of any commercial or financial relationships that could be construed as a potential conflict of interest.

## Publisher’s note

All claims expressed in this article are solely those of the authors and do not necessarily represent those of their affiliated organizations, or those of the publisher, the editors and the reviewers. Any product that may be evaluated in this article, or claim that may be made by its manufacturer, is not guaranteed or endorsed by the publisher.

## Footnotes

^1^ https://github.com/faylward/ncldv_markersearch

^2^ https://www.R-project.org

^3^ http://odv.awi.de
